# The relationship between lignin peroxidase and manganese peroxidase production capacities and cultivation periods of mushrooms

**DOI:** 10.1111/j.1751-7915.2012.00365.x

**Published:** 2012-09-11

**Authors:** Jian Z Xu, Jun L Zhang, Kai H Hu, Wei G Zhang

**Affiliations:** 1 School of Life Sciences, Fujian Agriculture and Forestry UniversityFuZhou, 350002, China; 2 School of Biotechnology, Jiangnan UniversityWuXi, 214122, China

## Abstract

Mushrooms are able to secrete lignin peroxidase (LiP) and manganese peroxidase (MnP), and able to use the cellulose as sources of carbon. This article focuses on the relation between peroxidase-secreting capacity and cultivation period of mushrooms with non-laccase activity. Methylene blue and methyl catechol qualitative assay and spectrophotometry quantitative assay show LiP secreting unvaryingly accompanies the MnP secreting in mushroom strains. The growth rates of hyphae are detected by detecting the dry hyphal mass. We link the peroxidase activities to growth rate of mushrooms and then probe into the relationship between them. The results show that there are close relationships between LiP- and/or MnP-secretory capacities and the cultivation periods of mushrooms. The strains with high LiP and MnP activities have short cultivation periods. However, those strains have long cultivation periods because of the low levels of secreted LiP and/or MnP, even no detectable LiP and/or MnP activity. This study provides the first evidence on the imitate relation between the level of secreted LiP and MnP activities and cultivation periods of mushrooms with non-laccase activity. Our study has significantly increased the understanding of the role of LiP and MnP in the growth and development of mushrooms with non-laccase activity.

## Introduction

Lignin peroxidase (LiP) and manganese peroxidase (MnP) are extracellular haem protein peroxidases (Shin *et al*., 2005; Sharma *et al*., 2011). Lignin peroxidase and MnP have been identified in fungal species and have been characterized at the molecular level (Conesa *et al*., 2002; Miki *et al*., 2011). Lignin peroxidase activity can be monitored by cleavage or H_2_O_2_-dependent oxidation of a wide variety of non-phenolic lignin model compounds, β-*O*-4-linked lignin model compounds, other methoxybenzenes, aromatic ring cleavages and C_α_-C_β_ cleavages (Archibald, 1992; Ikehata *et al*., 2004). It catalyses the aromatic polymer lignin and a variety of non-phenolic lignin model compounds in the presence of H_2_O_2_ to homologus aldehydes or ketones, and hydroxylation of benzylic methylene groups (Ikehata *et al*., 2004). Meanwhile, Singh and colleagues (2011) reported that MnP has the properties of both an oxidase and a peroxidase. It not only catalyse the lignin and phenolic lignin model compounds, but also catalyse the non-phenolic ones in the presence of certain compounds to polycyclic aromatic hydrocarbons by oxidation of Mn^2+^ to Mn^3+^ with H_2_O_2_ as an oxidant (Steffen *et al*., 2003; Shin *et al*., 2005). Lignin peroxidase and MnP are generally viewed as primary enzymes for degradation of lignin.

Mushroom cultivations have a very long history in China, about 1500 years. As science and technology have been developing so fast, more and more mushroom species are commercially produced. With the requirements of mushroom increasing year by year, the annual production is steadily increasing. Mushrooms are not only a good source of nutrients and vitamins (Mattila *et al*., 2001), but also a good resource of bioactive compounds (Sánchez, 2004). Wasser (2002) has reported that fungal polysaccharides are the best-known mushroom substances possessing antitumour and immunomodulating properties. Mushrooms are saprophytic basidiomycetes, mostly belong to wood-degrading white-rot or litter-degrading fungi (Lankinen, 2004). So far, mushroom cultivation is the most profitable way of utilizing lignocellulose-containing waste material (Zhang, 2003). Lignin, carbohydrates and organic and inorganic nitrogen sources are the major components of mushroom composts (Bonnen *et al*., 1994). Although some studies have reported that lignin is not a growth substrate for mushrooms (Kirk and Farrell, 1987), mushrooms have the ability to completely degrade the lignin to the cellulose molecule using LiP, MnP and laccase, and mushrooms can use the cellulose fraction as a source of carbon (Lara *et al*., 2003). Therefore, lignin can be used as the components of mushrooms, and the level of secreted laccase, LiP and MnP activities in the mushrooms would affect the growing cycles of mushrooms. Previous reports show that the mushroom strains with high level of secreted laccase activity have short growing cycles (Sun *et al*., 2011; Xu *et al*., 2012). And some reports indicate that fungal laccase partake detoxification of phenolic compounds and sporophore development (Bollag *et al*., 1988; Zhao and Kwan, 1999; Ohga and Royse, 2001). However, not all of mushroom strains are able to secrete laccase (Sun *et al*., 2011), and the role of laccase in biodegradation of lignin has not been well established (Bonnen *et al*., 1994). From the analyses above, we conclude that the levels of secreted LiP and MnP activities are closely related to the cultivation periods of mushrooms strains with non-laccase activities. To our knowledge, studies on the relation between these enzymes and growing cycles of mushrooms have not been investigated and reported.

The aim of this paper is to demonstrate the intimate relationship between the level of secreted LiP and MnP activities in the mushrooms and the cultivation periods of mushrooms. The results will be conducive to know more about the physiological feature of mushrooms, and also lay definite theoretical foundation for breeding high peroxidase-secreting strains that shorten artificial cultivation period of mushrooms.

## Results

### Methylene blue and methyl catechol qualitative assay

**Figure 1:**
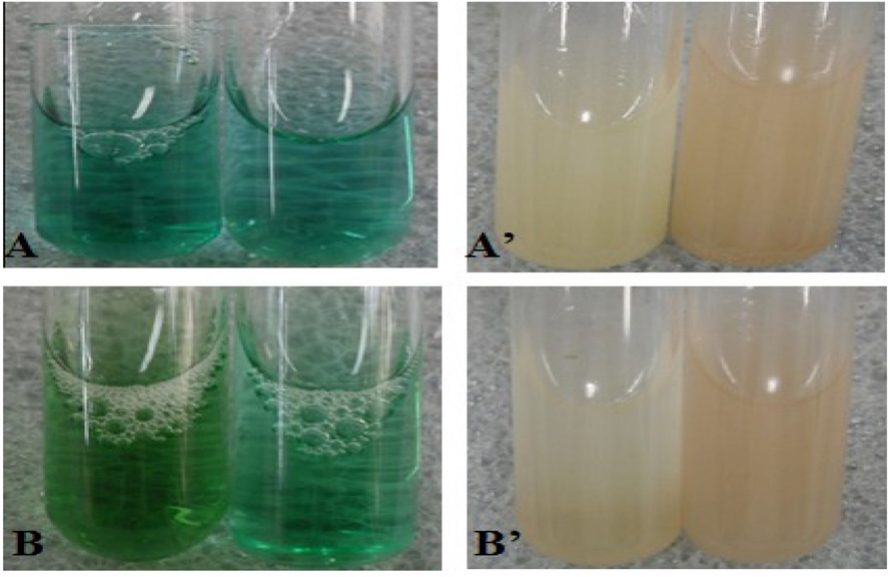
The qualitative assay of LiP and MnP after 7 days incubated in MYPGB at 24°C and pH 7.0 with shaking (100 r min^−1^). (A) Methylene blue qualitative assay of LiP from *G. frondosa*; (A′) methyl catechol qualitative assay of MnP from *G. frondosa*; (B) methylene blue qualitative assay of LiP from *Agrocybe* sp.; (B′) methyl catechol qualitative assay of MnP from *Agrocybe* sp. The left test tubes were control tests.

Peroxidase production capacities of different mushroom strains were compared and analysed by the colour change of mixture. The methylene blue and methyl catechol were used for a visual inspection for the LiP and MnP presence in the mixture respectively. The results are presented in Table 1 and Fig. 1. Ten different mushroom strains were divided into three groups based on peroxidase-secreting capacity. The colour change immediately shows high level of LiP or MnP production capacity. These strains including *Stropharia rugoso*, *Hypsizigus marmoreus*, *Tricholoma lobyense*, *Agrocybe cylindracea*, *Agrocybe* sp. and *Fistulina hepatica* could secrete LiP and MnP, which belonged to group I. *Pleurotus nebrodeusis* and *Grifola frondosa* could only secrete MnP, which belonged to group II. The other strains including *Pholiota nameko* and *Lepista irina* did not change the mixture colour after 1 h. Therefore, LiP and MnP were not detected in these strains, which belonged to group III. The strains in group I and group II also showed different levels of enzyme activities. The cultivation period from inoculations to the first time harvesting in solid-state systems showed great difference among 10 strains. The cultivation periods of those strains in group I were shown generally shorter than the other groups. Group II had middle level of cultivation periods, and group III had the longest cultivation periods among all groups. The strains in group I, group II or group III also showed different cultivation periods (Pang *et al*., 2003; Zhang, 2003; Sun *et al*., 2011). The results showed that LiP-secreting and MnP-secreting capacity and variety of mushroom are closely related to cultivation period. The mushroom strains have short cultivation periods because they can secrete LiP and MnP, and have high levels of enzyme activity. However, those other strains require long growing cycles due to the unitary enzyme and the low levels of enzyme activity, even no detectable enzyme activity. The results also indicated that the methylene blue reaction and the methyl catechol reaction can be used as rapid assay for visual inspection for the enzyme presence and capacity in the culture supernatant.

**Table 1:** The mushroom strains and their lignin-degrading peroxidase after 7 days incubated in MYPGB at 24°C and pH 7.0 with shaking (100 r min^−1^) and cultivation period

Strains	Strains' presentation no.	Decay type	LiP assay^a^	MnP assay^b^	Cultivation period^b^
*Stropharia rugoso*	ACCC51711	LDFs	+	+++	80–120^[1]^
*Hypsizigus marmoreus*	ACCC51622	WRFs	++	++	110–120^[1]^
*Tricholoma lobyense*	ACCC50754	LDFs	++	+++	80–100^[2]^
*Agrocybe cylindracea*	ACCC50913	WRFs	++	+++	90–100^[1]^
*Agrocybe* sp.	ACCC51110	LDFs	+	+++	80–90^[3]^
*Fistulina hepatica*	ACCC50672	WRFs	+	++	110–120^[3]^
*Pleurotus nebrodeusis*	ACCC51914	WRFs	−	+	100–150^[2]^
*Grifola frondosa*	ACCC50887	WRFs	−	+++	90–110^[1]^
*Pholiota nameko*	ACCC50331	WRFs	−	−	120–140^[2]^
*Lepista irina*	ACCC52235	LDFs	−	−	180–200^[3]^

a −: colour of mixture has no change; +, ++, +++: colour of mixture is changed more and more quickly.

b References [1], [2] and [3] are Sun *et al*., 2011, Zhang, 2003 and Pang *et al*., 2003 respectively, and the cultivation periods refer to the industrial production.

ACCC, Agricultural Culture Collection of China; LDFs, litter-degrading fungi; WRFs, white-rot fungi.

### Determination of LiP and MnP activity, hyphal dry weight and hyphal growth rate

**Figure 2:**
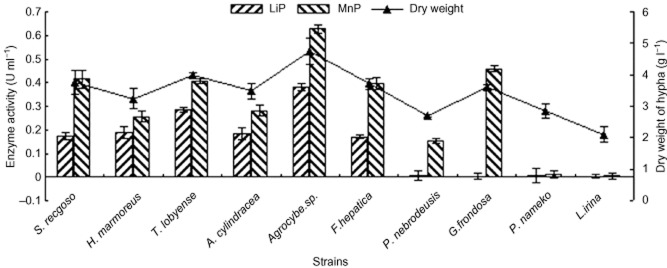
The LiP and MnP activities and the dry weights of 10 strains after 7 days incubated in MYPGB at 24°C and pH 7.0 with shaking (100 r min^−1^). Each value represents mean with standard error of three replicative experiments. The standard errors are shown as bars.

The LiP and MnP activities in culture supernatants of 10 strains at the seventh day are show in Fig. 2 (shown as bar graph). Both LiP and MnP were detected in group I by spectrophotometry. Only MnP was obviously detected in group II, and both LiP and MnP were difficult to detect in group III. The results of quantitative analysis were coincident with the results of qualitative analysis. Among these 10 strains, *Agrocybe* sp. showed the highest level of LiP and MnP activities, nearly 0.382 ± 0.016 U ml^−1^ and 0.627 ± 0.016 U ml^−1^ respectively. However, the LiP activities of *P. nebrodeusis*, *G. frondosa*, *P. nameko* and *L. irina* were extraordinary low, nearly 0.005 ± 0.021 U ml^−1^, 0.003 ± 0.012 U ml^−1^, 0.006 ± 0.029 U ml^−1^ and 0.002 ± 0.008 U ml^−1^ respectively. The MnP activities of *P. nameko* and *L. irina* were also very low, nearly 0.01 ± 0.016 U ml^−1^ and 0.005 ± 0.013 U ml^−1^ respectively. The activities of MnP were higher than the activities of LiP for every mushroom strain (Fig. 2).

**Figure 3:**
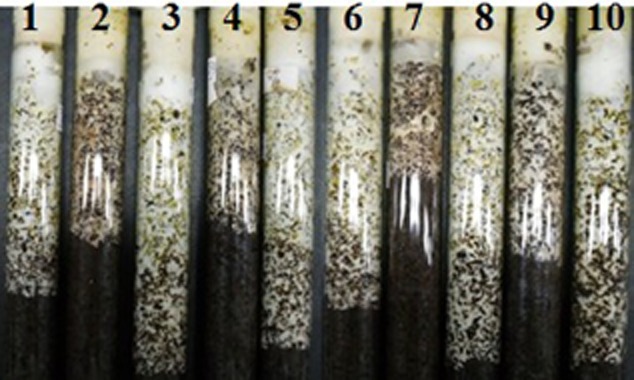
The hyphal growth rate of 10 strains. All strains were incubated in cotton-seed-hull test tube medium at 24°C for 14 days. (1) *A. cylindracea*; (2) *P. nameko*; (3) *Agrocybe* sp.; (4) *P. nebrodeusis*; (5) *F. hepatica*; (6) *G. frondosa*; (7) *L. irina*; (8) *T. lobyense*; (9) *H. marmoreus*; (10) *S. rugoso*.

The hyphal dry weights of every strain are also shown in Fig. 2 (shown as line graph). The dry weight of hypha reached 4.746 ± 0.41 g l^−1^ for *Agrocybe* sp. as the maximum and 2.107 ± 0.25 g l^−1^ for *L. irina* as the minimum. The results of hyphal dry weight were marched with the variety and activity of enzyme. In other word, the more the varieties of enzyme and the higher the activities of enzyme, the higher the dry weight of hypha. The hyphal growth rates of every strain are shown in Fig. 3. From this figure, we can realize that the results were marched with the upper experimental results. The results showed that LiP-secreting and MnP-secreting capacity and variety of mushroom are closely related to the speed of hyphal growth. The strains with LiP and MnP and the high-level enzyme activities showed the fast growth rate of hypha. The fast growth rate of hypha means this mushroom strain has short cultivation period. Therefore, these results are in accordance with the results of qualitative assay and previous reports (Pang *et al*., 2003; Zhang, 2003; Sun *et al*., 2011).

## Discussion

In this paper, we describe the intimate relationship between the level of secreted LiP and MnP and the cultivation periods of mushrooms with non-laccase activity. Although previous studies comparing the growing cycles of different mushroom strains (Sun *et al*., 2011) had shown that extracellular ligninolytic enzyme (laccase) had important role in the growth of mushrooms, not all of mushroom strains could secrete laccase (Xu *et al*., 2012). Previous reports had shown that the role of laccase in biodegradation of lignin has not been well established (Bonnen *et al*., 1994), and the other extracellular ligninolytic enzymes (LiP and MnP) are primary enzymes for degradation of lignin (Maeda *et al*., 2001). Meanwhile, much literature also reported that LiP and MnP have been detected in mushrooms (Leatham and Kirk, 1983; Bourbonnais and Paice, 1988; Bonnen *et al*., 1994; Cohen *et al*., 2001; Nagai *et al*., 2002). Although there are many reports about LiP and MnP from mushrooms, the results of the present work firstly confirm that the levels of secreted LiP and MnP are closely related to the cultivation periods of mushrooms strains with non-laccase activities.

Ten mushroom strains were divided into three groups according to the results of methylene blue and methyl catechol qualitative assay. Strains in group I was able to secrete LiP and MnP. Strains in group II was able to only secrete MnP, and strains in group III were not able to secrete LiP and MnP. These results were accordant with the results of the determination of LiP and MnP by spectrophotometry. Interestingly, there were no mushroom strains being able to only secret LiP. Previous reports had also shown that MnP appears to be more common than LiP (Orth *et al*., 1993; Hatakka, 1994; Vares and Hatakka, 1997). To our knowledge, there were no reports that mushroom strain could only secret LiP. All of that means the LiP secreting unvaryingly accompanies the MnP secreting in mushroom strains. The genes of LiP and MnP in the mushroom strains were linked genes, and this fact could be used to explain this results (Larraya *et al*., 1999; Doddapaneni *et al*., 2005).

The results of qualitative assay, quantitative assay and hyphal growth rate showed that many kinds of peroxidase and high level of LiP and/or MnP secreted by mushroom strains had short cultivation period, and had high growth rate of hyphae. The strains in group I were able to secrete LiP and MnP, and the cultivation periods of group I were shown generally shorter than the other groups. However, the strains in group III were not able to secrete LiP and MnP, and the strains in this group had longer cultivation periods than the other groups. Many reports have shown that LiP and MnP have a significant role in the degradation lignin to the cellulose molecule (Bonnen *et al*., 1994; Maeda *et al*., 2001; Conesa *et al*., 2002; Lara *et al*., 2003), and mushrooms can use the cellulose fraction as a source of carbon (Lara *et al*., 2003). Lankinen (2004) reported that combinations of extracellular ligninolytic enzymes (LiP, MnP and laccase) are important for lignin degradation. Saeki and colleagues (2011) also reported that the expression and properties of the enzymes will influence mycelia growth and fruit body development. Our findings were greatly supported by these previous investigations. Interestingly, the levels of LiP and MnP activities of *P. nameko* were negligible, 0.006 ± 0.029 U ml^−1^ and 0.01 ± 0.016 U ml^−1^ respectively, but the cultivation period and growth rate of *P. nameko* were higher than that of *P. nebrodeusis*, 2.852 ± 0.23 g l^−1^ and 2.694 ± 0.05 g l^−1^ respectively. The LiP and MnP activities of *P. nebrodeusis* were 0.005 ± 0.021 U ml^−1^ and 0.154 ± 0.009 U ml^−1^ respectively. Hatakka (2005) and Malviya and colleagues (2011) reported that polyphenol oxidase (PPO), endocellulase, glyoxal oxidase (GLOX) and aryl alcohol oxidase (AAO) produced by white-rot fungi play a role in the degradation of lignin. Previous reports have shown that *P. nameko* could secrete PPO, GLOX and AAO (Lankinen, 2004; Flurkey and Inlow, 2008). The higher level of PPO, GLOX and AAO activities of *P. nameko* than that of *P. nebrodeusis* might lead to the higher growth rate of *P. nameko* than that of *P. nebrodeusis*.

## Conclusions

In conclusion, we have studied LiP- and MnP-secreting capacity and activity from 10 industrially important edible mushrooms with non-laccase activity, and demonstrated a correlation between the kinds of peroxidase along with the levels of peroxidase activities and cultivation periods of 10 strains. The results could help us to know more about the physiological features of mushrooms. That is also an advantage of using them as theoretical foundation for breeding high peroxidase-secreting strains to improve the strain characteristics and shorten artificial cultivation period of edible mushrooms.

## Experimental procedures

### Mushroom strains and media

Ten strains were obtained from Fujian General Station of Technology Popularization for Edible Fungi (Fuzhou, China; Table 1) and maintained on potato–dextrose–agar medium (PDA; potato 200 g l^−1^, 20 g of glucose, 3 g of KH_2_PO_4_, 1.5 g of MgSO_4_·7H_2_O and 20 g of agar in 1000 ml of potato extract) at 25°C with periodic transfer. Potato extract was prepared by boiling 200 g of potato and filtering via eightfold gauze. Laccase activity was not detected in these strains (J. Xu and K. Hu, unpubished data). For peroxidase-secreting studies, all strains used in this study were grown in malt–yeast extract–peptone–glucose–bran extract (MYPGB) liquid medium (2.5 g of malt extract, 1.0 g of yeast extract, 1.0 g of peptone and 5.0 g of glucose in 1000 ml of wheat brand extract). The preparation of sweat bran extract was carried out based on the protocol of Saeki and colleagues (2011) with the following modification. Ten grams of wheat bran (*Triticum aestivum*, strain zhongyou 206) in 1000 ml of distilled water was autoclaved for 15 min and filtered by vacuum filter (filter disc diameter: *Φ* 120 mm).

### Preparation of culture supernatants

Ten mycelial blocks (10 mm diameters) were placed in a 250 ml Erlenmeyer flask containing 100 ml of MYPGB liquid medium, and incubated at 24°C with shaking (100 r min^−1^) for 7 days. The culture supernatants were collected by centrifugation (10 min at 10 000 *g*) after cultivation.

### Qualitative assay of peroxidase

Lignin peroxidase was qualitatively assayed according to Magalhães and colleagues (1996). The colour that develops in the presence of LiP was compared with a blank assay where inactivated culture supernatants were used to replace activated culture supernatants. Mixtures with LiP show a change in the blueness from a greenish blue to purple blue. The speed of colour change has a direct connection with the LiP activity.

The protocol of MnP assay is as follows: 2 ml of culture supernatants were added to the reaction mixture (total volume, 3 ml) consisting of 0.4 mmol l^−1^ methyl catechol, 0.2 mmol l^−1^ MnSO_4_ and 50 mmol l^−1^ sodium succinate buffer solution (pH 4.5). Reactions were initiated by the addition of H_2_O_2_ to a final concentration of 0.1 mmol l^−1^ (Brown *et al*., 1993). The inactivated culture supernatants were used in blank assay. Mixtures with MnP show a change from buff to tawny. The speed of colour change has a direct connection with the MnP activity.

### Quantitative assay of peroxidase

All of enzyme assays were performed at room temperature with culture supernatants. Results are reported as means of three separate assays.

Lignin peroxidase activity was measured according to Maeda and colleagues (2001). The reaction mixture contained 20 mmol l^−1^ sodium succinate (pH 3), 0.5 mmol l^−1^ veratryl alcohol and 0.1 mmol l^−1^ H_2_O_2_. After adding 100 μl of culture supernatants to 3 ml of the reaction mixture, the rate of oxidation of veratryl alcohol to veratraldehyde was monitored by measuring the rate of increase in absorbance at 310 nm. The culture supernatants boiled for 10 min was used in the control.

Manganese peroxidase activity was performed based on the protocol of Wariishi and colleagues (1992) with the following modification. The total volume of reaction is 3 ml, and culture supernatants boiled for 10 min was used in the control.

### Determination of the hyphal growth rate

As inocula, mycelia blocks (15 mm diameters) from strains were subcultured in cotton-seed-hull test tube (specification and size: 25 mm × 180 mm) and incubated at 24°C for 14 days. The cultivation materials of cotton-seed-hull were prepared as the method of Xu and colleagues (2012). The mass of cultivation material was 60 ± 0.5 g each test tube.

### Dry hyphae mass

After preparation of culture supernatants, the remaining cenobium of each strain was dried at 80°C overnight in a drying cabinet and then weighed.

## Conflict of interest

None declared.
